# Circadian expression of *Fabp7* mRNA is disrupted in *Bmal1* KO mice

**DOI:** 10.1186/s13041-020-00568-7

**Published:** 2020-02-24

**Authors:** Jason R. Gerstner, Georgios K. Paschos

**Affiliations:** 1 Elson S. Floyd College of Medicine, Washington State University, Spokane, WA 99202 USA; 2 Sleep and Performance Research Center, Washington State University, Spokane, WA 99202 USA; 3 Steve Gleason Institute for Neuroscience, Washington State University, Spokane, WA 99202 USA; 4 Institute for Translational Medicine and Therapeutics, University of Pennsylvania, Philadelphia, PA 19104 USA

## Abstract

The astrocyte brain-type fatty acid binding protein (*Fabp7*) gene expression cycles globally throughout mammalian brain, and is known to regulate sleep in multiple species, including humans. The mechanisms that control circadian *Fabp7* gene expression are not completely understood and may include core circadian clock components. Here we examined the circadian expression of *Fabp7* mRNA in the hypothalamus of core clock gene *Bmal1* knock-out (KO) mice. We observed that the circadian rhythm of *Fabp7* mRNA expression is blunted, while overall *Fabp7* mRNA levels are significantly higher in *Bmal1* KO compared to control (C57BL/6 J) mice. We did not observe any significant changes in levels of hypothalamic mRNA expression of *Fabp3* or *Fabp5*, two other fatty acid binding proteins expressed in mammalian brain, between *Bmal1* KO and control mice. These results suggest that *Fabp7* gene expression is regulated by circadian processes and may represent a molecular link controlling the circadian timing of sleep with sleep behavior.

## Main text

Sleep behavior is exhibited by virtually every species studied, while the precise function of sleep remains unknown. Understanding the underlying cellular and molecular mechanisms that relay sleep behavior may resolve important clues as to sleep function. Sleep is thought to be governed by two processes, a circadian system, which controls the daily timing of sleep, and a homeostatic system, which regulates sleepiness based on previous time spent awake [1, 2]. Circadian rhythms are regulated by a well-defined and phylogenetically conserved transcriptional/translational autoregulatory negative feedback loop [3], which includes the core clock gene *Bmal1* [4]. Bmal1 is a basic helix-loop-helix transcription factor known to heterodimerize with core circadian factors CLOCK or NPAS2 and bind to E-box elements in the promoter of downstream target genes to influence circadian their transcriptional output and behavior [5, 6]. Deletion of *Bmal1* is the only single gene deletion that disrupts circadian clock function, whereas single gene deletion of other clock components only leads to attenuated circadian rhythms [7]. However, exactly how this circadian clock shapes molecular events that in turn regulate sleep behavior are not well understood.

Fatty-acid binding proteins (Fabp) comprise a family of small (~ 15 kDa) hydrophobic ligand binding carriers with high affinity for long-chain fatty-acids for intracellular transport, and are associated with metabolic, inflammatory, and energy homeostasis pathways [8, 9]. These include three that are expressed in the adult mammalian central nervous system (CNS), and are Fabp3 (H-Fabp), Fabp5 (E-Fabp), and Fabp7 (B-Fabp). Fabp3 is predominantly expressed in neurons, Fabp5 is expressed in multiple cell types, including both neurons and glia, and Fabp7 is enriched in astrocytes and neural progenitors. *Fabp7* mRNA was identified as a unique transcript elevated in multiple hypothalamic brain regions of mice during the sleep phase [10], and Fabp7 is also known to regulate sleep in flies, mice, and humans [11]. Here we were interested in determining whether circadian *Fabp7* mRNA expression is disrupted in mice that lack the core circadian clock gene *Bmal1*.

**Fig. 1:**
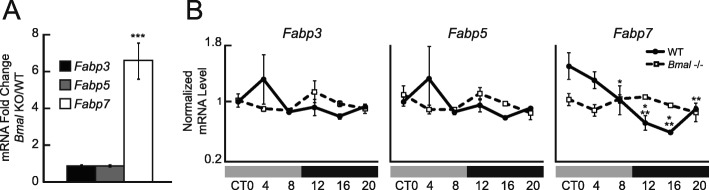
Increased baseline *Fabp7* mRNA expression, and disruption of its circadian rhythm, in *Bmal1* KO. (**a**) Average expression of hypothalamic *Fabp7* mRNA is ~7fold greater in BMAL KO compared to WT mice, while *Fabp3* mRNA and *Fabp5* mRNA are stable (*n* = 18 per group). ***p < 0.001 *Fabp7* mRNA KO/WT vs. *Fabp3* mRNA or *Fabp5* mRNA KO/WT (t-test). (**b**) Normalized mRNA expression values (to mean of circadian values within each group) of various *Fabps* depicts the circadian oscillation of *Fabp7* mRNA in C57BL/6 WT which is absent in BMAL KO mice (*n* = 3 per group per timepoint). *Fabp7* mRNA shows a significant circadian dependent change in expression based on genotype. Two-way ANOVA (*p* < 0.001). ***p < 0.001, **p < 0.01, *p < 0.05 vs ZT0 (post-hoc Bonferroni), while there is no significant difference for circadian variation of *Fabp7* mRNA in BMAL KO mice

To test whether circadian *Fabp7* mRNA expression is regulated by the core molecular clock, we compared *Fabp7* transcript levels in hypothalamus of *Bmal1* KO to wild-type (WT) littermate control C57BL/6 J mice using qPCR analysis. We observed that baseline levels of *Fabp7* mRNA expression in the *Bmal1* KO mouse were higher compared to WT littermate controls, irrespective of circadian time (Fig. 1a, Fig. S1), while the circadian expression of *Fabp7* mRNA was completely ablated in *Bmal1* KO mice compared to controls (Fig. 1b). In order to test whether other CNS expressing *Fabps* have altered mRNA expression as a result of *Bmal1* deficiency, we profiled *Fabp3* and *Fabp5* and found that neither the baseline expression or circadian pattern of these transcripts were affected by *Bmal1* (Fig. 1a, b), suggesting that among Fabps expressed in the CNS, both the baseline and circadian profile of transcription affected by the core clock is *Fabp7* specific.

Circadian *Fabp7* mRNA expression has been shown to be regulated by the nuclear hormone receptor Rev-erbα (NR1D1), a transcriptional repressor, via Rev-erbα response elements (ROREs) in the *Fabp7* gene promoter [12]. Since *Bmal1* deficiency greatly suppresses Rev-erbα expression, it is likely that the circadian regulation of *Fabp7* mRNA by Bmal1 is through Rev-erbα. Studies determining whether Bmal1 regulates *Fabp7* mRNA indirectly via Rev-erbα or whether the *Fabp7* gene promoter also has functional E-box binding sites will be crucial for identifying the clock-regulated expression of this gene. *Fabp7* mRNA expression was also shown to be upregulated in mice with deletion of *Bmal1* in neurons and astrocytes [13]. Future studies using cell-specific knock-down of *Bmal1* to determine whether disruption of core clock genes in astrocytes regulates circadian *Fabp7* mRNA expression will be important for our understanding of how clock-controlled genes in non-neural cells regulate pathways affecting behavior. For example, recent studies show that astrocytes are able to regulate daily rhythms in the master circadian pacemaker of the hypothalamus, the suprachiasmatic nucleus (SCN), and circadian behavior [14, 15]. Further, post-transcriptional processing mechanisms known to operate on *Fabp7* mRNA targeting to perisynaptic processes [16] will be important to characterize following circadian clock disruption in astrocytes. Determining whether circadian *Fabp7* expression is necessary for daily rhythms in SCN and extra-SCN physiology [17] and related behavior are additional avenues for future study.

## Supplementary information

**Additional file 1.**

**Additional file 2.**

## Data Availability

All data generated or analyzed during this study are included in this published article.
