# Supplementary material for: Circadian expression of Fabp7 mRNA is disrupted in Bmal1 KO mice
Source: Mol Brain. 2020 Feb 24;13:26. doi: 10.1186/s13041-020-00568-7 (PMC7041087; doi:10.1186/s13041-020-00568-7)
Supplement: Supplementary file 1 — Additional file 1. [file 13041_2020_568_MOESM1_ESM.pdf]

Figure S1

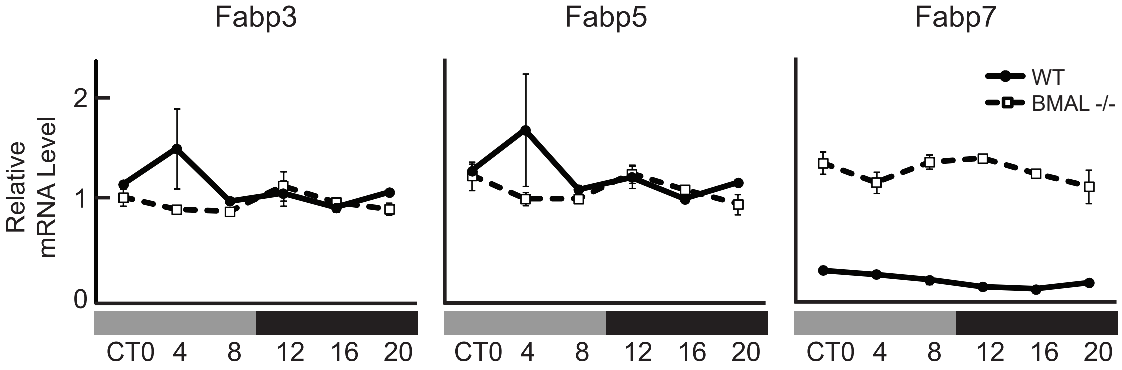

Average expression of Fabp7 mRNA is ~7fold greater in BMAL KO compared to WT, while Fabp3 and Fabp5 are stable. Relative mRNA expression of various Fabps in BMAL KO vs. C57BL/6 WT mice in constant dark conditions. Values represent relative mRNA level to Gapdh as measured by qRT-PCR.
