# Supplementary material for: Circadian expression of Fabp7 mRNA is disrupted in Bmal1 KO mice
Source: Mol Brain. 2020 Feb 24;13:26. doi: 10.1186/s13041-020-00568-7 (PMC7041087; doi:10.1186/s13041-020-00568-7)
Supplement: Supplementary file 2 — Additional file 2. [file 13041_2020_568_MOESM2_ESM.pdf]

Methods

Animals

Bmal1 KO mice were obtained from C. Bradfield (University of Wisconsin, Madison, WI). Control mice used in this study were WT littermate controls to the *Bmal1* KO mice and were generated by in house breeding. Male animals (6-10 week old) were entrained to a 12 hour light, 12 hour dark light cycle for a minimum of 2 weeks prior to release into constant darkness for harvesting brain tissue (under dim red light). All animals in this study were housed according to the guidelines of the Institutional Animal Care and Use Committee (IACUC) of the University of Pennsylvania. All experimental protocols were approved by IACUC.

qPCR

Total RNA from male brain hypothalamus was isolated using the Qiagen RNeasy Kit. Hypothalamic sections were taken from freshly isolated brains with five cuts made using a razor blade. Viewing the ventral side of the brain, two coronal cuts were placed at the apex of the optic chiasm and the rostral margin of the mammillary bodies. This slab was then placed flat, and two cuts were placed on either side of the optic chiasm. A third cut was placed just above the third ventricle. All cuts from brains were approximately the same size. Reverse transcription was performed using an RNA-cDNA kit (Applied Biosystems). Real-time PCR was performed using ABI TaqMan primers and reagents on an ABI Prism 7500 thermocycler according to the manufacturer's instructions. All mRNA measurements were normalized to *Gapdh* mRNA levels. Average expression of hypothalamic *Fabp3*, *Fabp5* and *Fabp7* mRNA was calculated across all 6 circadian timepoints and a ratio calculated (KO/WT) for values depicted in Figure 1A.

Primers:

Fabp3 Forward CTG-ACT-CTC-ACT-CAT-GGC-AGT-GT

Fabp3 Reverse GCC-AGG-TCA-CGC-CTC-CTT

Fabp5 Forward CGA-CAG-CTG-ATG-GCA-GAA-AAA

Fabp5 Reverse GAC-CAG-GGC-ACC-GTC-TTG

Fabp7 Forward CTC-TGG-GCG-TGG-GCT-TT

Fabp7 Reverse TTC-CTG-ACT-GAT-AAT-CAC-AGT-TGG-TT

Gapdh Forward primer CAT-GGC-CTT-CCG-TGT-TCC-T

Gapdh Reverse primer GCG-GCA-CGT-CAG-ATC-CA

Data analysis

Statistical analysis included student's t-test, two-way ANOVA, and Bonferroni post-hoc analysis GraphPad Prism software was used for the analysis. Sample sizes were based on variability of the test measurement and the desire to detect a minimal 10% difference in the variables assessed with $\alpha = 0.05$ and power $(1-\beta) = 0.8$.
